# Development and Study of Hydrophilic Ointment Compositions with a Dextrin/Polyvinyl Alcohol/Iodine Complex (D/PVA/I)

**DOI:** 10.3390/ph19060969

**Published:** 2026-06-22

**Authors:** Zhassur Taganov, Anel Azamatova, Roza Karzhaubayeva, Gulshat Baigaipova, Zhanar Iskakbayeva, Saltanat Jumabayeva, Ardak Jumagaziyeva, Ilya Korotetskiy, Lyudmila Ivanova, Natalya Zubenko, Seitzhan Turganbay, Amir Azembayev

**Affiliations:** 1 Scientific Center for Anti-Infectious Drugs JSC, Almaty 050060, Kazakhstan; taganovjasur@mail.ru (Z.T.);; 2 Faculty of Natural Sciences, Kazakh National Women’s Teacher Training University, Almaty 050000, Kazakhstan; turganbay.s@gmail.com; 3 “One Belt, One Road” Petroleum Engineering, Kazakh-British Technical University, Almaty 050000, Kazakhstan

**Keywords:** hydrophilic ointment compositions, polymer–iodine complex, polyethylene glycol, antimicrobial activity, cytotoxicity, triiodide ions, semisolid system

## Abstract

**Background:** Iodine-based antimicrobial systems remain highly attractive due to their broad-spectrum activity; however, the clinical application of free iodine is limited by its instability and cytotoxicity. This study aimed to develop polyethylene glycol (PEG)-based hydrophilic ointment formulations containing a dextrin/polyvinyl alcohol/iodine complex (D/PVA/I) and to evaluate their physicochemical properties, antimicrobial activity, and cytotoxicity. **Methods:** Hydrophilic ointment formulations containing 2.5%, 5.0%, and 10.0% D/PVA/I were prepared using a PEG-based matrix composed of PEG 4000, PEG 400, and glycerol. Physicochemical characterization included organoleptic evaluation, pH measurement, rheological analysis, and UV–visible (Ultraviolet–visible) spectroscopy. Antimicrobial activity was assessed using agar diffusion and minimum bactericidal concentration (MBC) assays against *Staphylococcus aureus*, *Escherichia coli*, *Enterococcus hirae*, and *Pseudomonas aeruginosa*. Cytotoxicity was evaluated in Madin–Darby Canine Kidney (MDCK) cells using the MTT assay. **Results:** All formulations exhibited homogeneous semisolid structure and physiologically acceptable pH values (4.94–5.45). Rheological analysis demonstrated non-Newtonian pseudoplastic (shear-thinning) behavior. The flow behavior index (n) ranged from 0.03 to 0.33 according to the Ostwald–de Waele model, confirming shear-thinning characteristics, while viscosity increased with increasing D/PVA/I concentration. UV–visible spectroscopy confirmed the presence of triiodide ions (I_3^−^_), characterized by absorption maxima at approximately 287 and 350 nm, indicating preservation of active iodine species within the PEG matrix, while placebo (blank) formulation analysis confirmed the absence of corresponding absorption bands, demonstrating that the PEG-based matrix does not contribute to the characteristic spectral features. The formulations demonstrated broad-spectrum antimicrobial activity, with MBC values ranging from 0.01 to 0.02 µg/mL. Cytotoxicity studies revealed moderate toxicity of the D/PVA/I complex (*CC*_50_ = 0.82%) (50% cytotoxic concentration (*CC*_50_) and significantly lower toxicity of the PEG-based ointment base (*CC*_50_ = 18.38%). **Conclusions:** The developed PEG-based hydrophilic ointment formulations containing the D/PVA/I complex demonstrated favorable physicochemical characteristics, stability of iodine species, pronounced antimicrobial activity, and acceptable cytotoxicity profiles. These findings highlight the potential for the developed systems to be promising topical antimicrobial formulations.

## 1. Introduction

The rapid spread of antimicrobial resistance (AMR) represents a major global health challenge, significantly reducing the effectiveness of existing therapeutic strategies and necessitating the development of alternative antimicrobial systems with improved efficacy and safety profiles [1,2]. The World Health Organization has identified AMR as one of the most critical threats to global public health, emphasizing the urgent need for non-antibiotic antimicrobial alternatives within global containment strategies [3]. In this context, iodine-based compounds remain highly attractive due to their broad-spectrum antimicrobial activity and nonspecific mechanism of action involving oxidative damage to proteins, nucleic acids, and cellular membranes of microorganisms [4,5]. Notably, topical antiseptics such as iodine-based formulations have regained significant attention within antimicrobial stewardship programs due to their broad-spectrum activity and favorable resistance profile [6,7]. However, the clinical application of molecular iodine is limited by its high volatility, chemical instability, and potential cytotoxic and irritant effects, which restrict its direct incorporation into pharmaceutical formulations [5,8].

To overcome these limitations, considerable attention has been directed toward the development of polymer–iodine complexes capable of stabilizing active iodine species and enabling controlled release [9,10,11]. These systems, broadly classified as iodophors, facilitate the sustained release of active iodine while minimizing the cytotoxic effects associated with free molecular iodine [12,13]. Polymer-encapsulated iodine delivery systems, in which iodine is stabilized within hydrophilic macromolecular matrices, have demonstrated significant potential in biomedical applications by modulating iodine availability, reducing volatility, and improving local tolerability [5,14,15]. In such systems, iodine predominantly exists in the form of polyiodide structures, particularly triiodide ions (I_3^−^_), generated through interactions between molecular iodine and iodide ions within a polymeric matrix. Stabilization of these species plays a critical role in determining both antimicrobial efficacy and biocompatibility [9,10]. Hydrophilic polymers, including polysaccharides and synthetic polymers such as polyvinyl alcohol (PVA), have demonstrated strong potential for forming supramolecular networks that modulate iodine release and improve system stability [16,17].

Recently, a dextrin/polyvinyl alcohol/iodine complex (D/PVA/I) was developed by our research group and demonstrated high aqueous solubility, a high proportion of triiodide ions, and pronounced antimicrobial activity against a broad range of microorganisms [15]. The combination of biodegradable dextrin and film-forming PVA provides a functional polymeric matrix capable of stabilizing iodine while maintaining acceptable biocompatibility. The biocompatibility of PVA-based matrices has been well documented in biomedical and pharmaceutical applications, supporting their suitability for tissue contacting formulations [18]. Nevertheless, despite promising characteristics of the active complex itself, its behavior within semisolid topical formulations remains insufficiently investigated.

The development of hydrophilic semisolid systems introduces additional factors that may influence formulation performance, including polymer matrix interactions, iodine speciation, rheological properties, and diffusion-controlled release [19,20,21]. In this context, recent studies have also explored the development of semisolid formulations incorporating natural bioactive compounds with antimicrobial activity. For instance, Mombekov et al. reported the development of an antifungal gel based on pomiferin isolated from *Maclura aurantiaca* fruits grown in Kazakhstan, where optimization of the solvent system (DMSO–PG–water, 1:4:1) and comprehensive rheological characterization were critical to achieving a stable thixotropic gel structure with favorable antifungal properties [22]. Polyethylene glycol (PEG)-based systems are widely used as hydrophilic ointment bases due to their biocompatibility, chemical inertness, and ability to provide reproducible release profiles. PEG-based hydrophilic ointments are recognized in major pharmacopeias as standard water-soluble semisolid vehicles offering excellent compatibility with a wide range of active pharmaceutical ingredients and favorable rheological properties for topical application [23,24]. Importantly, the interaction between PEG and molecular iodine is mediated by charge transfer complexation between the ether oxygen atoms of the polymer and I_2_ molecules. The oxygen atoms in the PEG structural repeat unit (–[O–CH_2_–CH_2_]_n_–) possess lone electron pairs that confer electron-donor properties, enabling them to coordinate iodine molecules, thereby inducing polarization and subsequent stabilization of iodine-containing anions [25,26,27]. The proposed mechanism involves several sequential stages: (i) formation of a donor–acceptor complex between the ether groups of PEG and the I_2_ molecule; (ii) polarization of the iodine molecule with partial charge transfer and generation of ion pairs; (iii) reaction of the iodide ion with an additional I_2_ molecule to form a stable triiodide anion (I_3^−^_); and (iv) stabilization of the system through electrostatic and van der Waals interactions within the polymeric matrix. Thus, PEG serves not merely as an inert vehicle but actively participates in the stabilization of reactive iodine species, enabling their controlled and prolonged release, reducing volatility, and potentially lowering cytotoxicity. However, the influence of PEG matrices on stabilization of polyiodide species and the relationship between physicochemical characteristics and biological activity remain insufficiently understood [21,22].

Cytotoxicity evaluation is an essential stage in the development of topical pharmaceutical systems, as it enables assessment of tissue compatibility and identification of safe concentration ranges. Determination of cytotoxic effects is particularly important for iodine-containing systems, where antimicrobial efficacy must be balanced with biocompatibility. Previous comparative studies have demonstrated that the cytotoxicity of iodine-containing formulations is strongly dependent on the carrier system and the concentration of free iodine, with polymer-based matrices exhibiting significantly improved safety profiles compared to aqueous iodine solutions [28,29].

Despite the growing number of studies on polymer–iodine systems, systematic investigations addressing the influence of D/PVA/I concentration on the physicochemical, rheological, antimicrobial, and cytotoxic properties of PEG-based semisolid formulations remain limited. In particular, preservation of triiodide ions within semisolid matrices and their relationship with biological activity remain insufficiently explored.

Therefore, the aim of this study was to develop PEG-based hydrophilic ointment formulations containing different concentrations of the D/PVA/I complex (2.5%, 5.0%, and 10.0%) and to evaluate their physicochemical properties, rheological behavior, iodine speciation, antimicrobial activity, and cytotoxicity. The obtained results provide insight into the structure–property–activity relationships governing polymer–iodine semisolid systems and demonstrate their potential for topical antimicrobial applications. This approach aligns with the growing emphasis on iodine-based antiseptics as effective alternatives to antibiotics in the management of superficial infections and wound colonization by resistant pathogens, including biofilm-forming and antiseptic-resistant species [30,31].

## 2. Results

### 2.1. Organoleptic Properties and Homogeneity

The organoleptic and physical characteristics of the developed PEG-based hydrophilic ointment compositions are presented in Table 1. All formulations exhibited a homogeneous semisolid structure without visible phase separation or foreign particles, indicating successful incorporation and uniform distribution of the D/PVA/I complex within the hydrophilic matrix [32].

A gradual color change from dark gray to black was observed with increasing concentration of the D/PVA/I complex, corresponding to the higher content of iodine-containing components in the formulations. All samples exhibited homogeneous semisolid consistency without visible phase separation or foreign inclusions and possessed a characteristic odor typical of iodine-containing systems. The obtained results indicate successful incorporation of the D/PVA/I complex into the PEG-based hydrophilic matrix and formation of physically stable semisolid systems suitable for further physicochemical and biological evaluation [32,33].

### 2.2. pH Analysis of Ointment Formulations

The pH values of the developed PEG-based hydrophilic ointment formulations are presented in Table 2. The pH ranged from 4.94 to 5.45, with a gradual decrease observed as the concentration of the D/PVA/I complex increased from 2.5% to 10.0%.

The decrease in pH may be associated with the presence of iodine-containing species and equilibrium processes involving iodide and triiodide ions within the formulations. Nevertheless, all samples remained within the physiologically acceptable pH range for topical semisolid systems and were considered compatible with the natural pH of human skin [34].

These findings indicate that incorporation of the D/PVA/I complex into the PEG-based matrix does not cause critical pH alterations and supports the suitability of the developed formulations for topical application.

### 2.3. Rheological Properties

**Figure 1:**
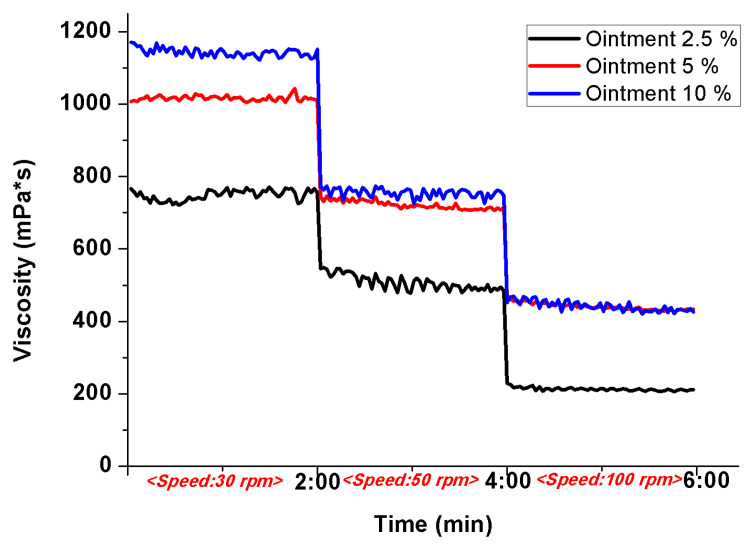
Viscosity of PEG-based hydrophilic ointment compositions containing the D/PVA/I complex (2.5%, 5.0%, and 10.0%) as a function of rotational speed (30–100 rpm).

The rheological behavior of the developed PEG-based hydrophilic semisolid formulations containing the D/PVA/I complex was evaluated at different rotational speeds; the results are presented in Figure 1. All formulations demonstrated a gradual decrease in viscosity with increasing rotational speed, indicating non-Newtonian pseudoplastic (shear-thinning) behavior characteristic of hydrophilic semisolid systems [35,36].

At a rotational speed of 30 rpm, the highest viscosity was observed for the formulation containing 10.0% D/PVA/I (~1143 mPa·s), followed by the 5.0% (~1016 mPa·s) and 2.5% (~749 mPa·s) formulations. With increasing rotational speed to 50 and 100 rpm, a decrease in viscosity was observed for all samples. This flow behavior confirms the pseudoplastic nature of the systems, typical for hydrophilic semisolid formulations intended for topical application.

**Figure 2:**
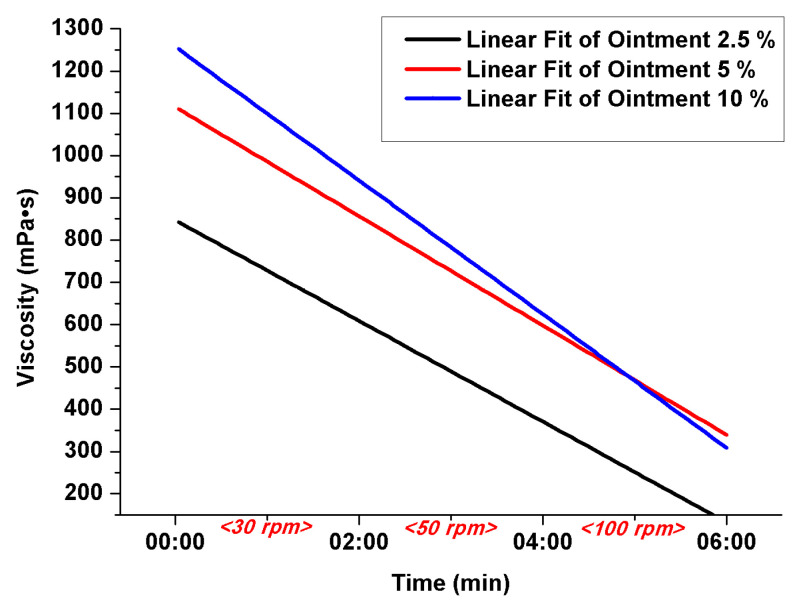
Linear regression of viscosity versus rotational speed (30–100 rpm) for PEG-based hydrophilic ointment compositions containing 2.5%, 5.0%, and 10.0% D/PVA/I.

The linear regression analysis of viscosity as a function of rotational speed is presented in Figure 2. It was observed that viscosity decreases consistently with increasing rotational speed, further confirming the pseudoplastic flow behavior of the studied compositions. At the same time, increasing the concentration of the D/PVA/I complex from 2.5% to 10.0% resulted in higher viscosity values, which may be attributed to enhanced structuring within the system.

The observed increase in viscosity with higher concentrations of the D/PVA/I complex may be associated with stronger intermolecular interactions and the formation of a more structured internal network within the semisolid matrix, leading to increased resistance to flow.

To provide a quantitative characterization of the flow behavior, the rheological data were further analyzed using the Ostwald–de Waele power-law model:

τ = Kγ^n^

where τ is the shear stress, γ is the shear rate, K is the consistency index, and n is the flow behavior index. The calculated rheological parameters are presented in Table 3. The n values were 0.03, 0.33, and 0.27 for formulations containing 2.5%, 5.0%, and 10.0% D/PVA/I, respectively. The corresponding K values were 2.43 × 10^4^, 1.13 × 10^4^, and 1.56 × 10^4^ mPa·s^n^. In all cases, n values were lower than 1, confirming the non-Newtonian pseudoplastic (shear-thinning) behavior of the developed semisolid systems. These findings quantitatively support the viscosity profiles observed in Figure 1 and Figure 2.

From a pharmaceutical perspective, the obtained rheological profile is characteristic of soft hydrophilic semisolid compositions. The decrease in viscosity with increasing rotational speed (shear-thinning behavior) facilitates ease of application and uniform spreading under mechanical stress. After the removal of mechanical stress, higher viscosity at lower rotational speeds may potentially contribute to retention at the site of application; however, further studies (e.g., adhesion and texture analysis) are required to confirm this assumption [37,38].

### 2.4. UV–Visible Spectral Analysis

UV–visible spectral measurements were performed in the wavelength range of 250–400 nm using a UV–visible spectrophotometer. Preliminary scanning below 220 nm revealed pronounced baseline instability and increased background absorption, likely associated with the polyethylene glycol (PEG) matrix and solvent interference at shorter wavelengths. Therefore, the lower scanning limit was set at 250 nm to ensure reliable spectral acquisition.

**Figure 3:**
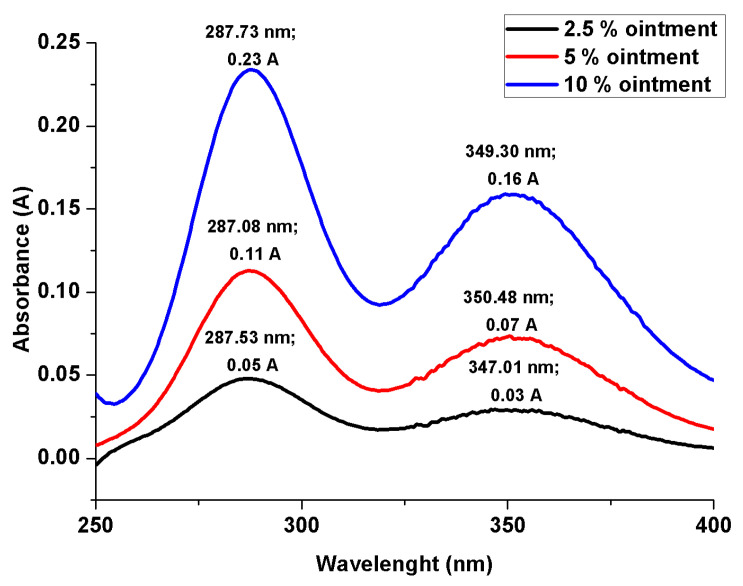
UV–visible absorption spectra of PEG-based hydrophilic ointment compositions containing 2.5%, 5.0%, and 10.0% D/PVA/I complex.

**Figure 4:**
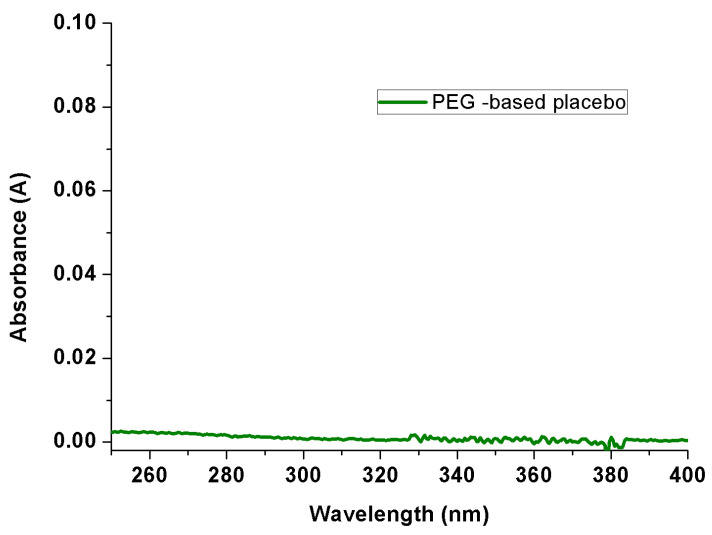
UV–visible spectrum of the placebo PEG-based hydrophilic ointment formulation without the iodine-containing active substance.

To evaluate the possible contribution of the PEG-based ointment matrix to the observed UV–visible absorption profile, the spectrum of a placebo formulation without the iodine-containing active substance was additionally investigated (Figure 3 and Figure 4). The placebo formulation demonstrated only weak low-intensity background absorption throughout the analyzed spectral range and did not exhibit distinct absorption maxima near 287 nm and 350 nm. Minor fluctuations and slight spectral noise were observed in the 327–384 nm region, indicating a limited influence of auxiliary PEG matrix components on the baseline profile.

The UV–visible spectra of PEG-based hydrophilic semisolid formulations containing the D/PVA/I complex exhibited two characteristic absorption maxima at approximately 287 nm and 350 nm (Figure 3). These absorption bands correspond to electronic transitions of triiodide ions (I_3^−^_) formed through interactions between iodide ions and molecular iodine within the polymeric matrix [39]. Slight spectral irregularities were also observed in the 327–384 nm region of formulations containing the active substance; however, the characteristic peaks at 287 nm and 350 nm remained clearly distinguishable in all formulations.

Quantitative spectral data, including λmax values and corresponding absorbance values, are presented in Table 4. Absorbance intensity increased proportionally with increasing concentration of the D/PVA/I complex (2.5%, 5.0%, and 10.0%), indicating concentration-dependent spectral behavior [40,41,42,43].

The consistency of λmax values among all formulations suggests preservation of the electronic structure of iodine-containing species following incorporation into the PEG matrix. The observed absorption maxima are consistent with previously reported spectral characteristics of D/PVA/I complexes, where absorption bands near 199 nm, 287 nm, and 351 nm were detected [15]. The absence of the absorption band near 199 nm in the present study can be explained by exclusion of the far UV region due to strong background absorption of the PEG matrix.

Overall, the obtained spectral data, together with placebo control analysis, confirm preservation of triiodide ions and stability of the iodine–iodide system within the developed semisolid formulations, while demonstrating that the PEG ointment matrix does not significantly contribute to the characteristic absorption maxima observed at 287 nm and 350 nm (Figure 4).

### 2.5. Determination of Triiodide Ion Concentration

The quantitative determination of triiodide ion (I_3^−^_) content in PEG-based hydrophilic ointment (semisolid) compositions containing the D/PVA/I complex was performed based on UV–visible spectroscopic data using the Beer–Lambert law [39,40,41,42,43]:

*A* = *ε⋅l⋅c* (1)

where *A* is the absorbance, *ε* is the molar extinction coefficient (L·mol^−1^·cm^−1^), *l* is the optical path length (cm), and *c* is the concentration of the absorbing species (mol/L). This relationship describes the linear dependence between absorbance and concentration of the analyte.

For the calculations, absorbance values at approximately 350 nm, corresponding to the absorption maximum of the triiodide ion, were used. The optical path length was 1 cm. The molar extinction coefficient for I_3^−^_ at this wavelength was taken from the literature as *ε* ≈ 2.6 × 10^4^ L·mol^−1^·cm^−1^ [39].

The concentration of triiodide ions was calculated using the following equation:

(2) $$c=\frac{A}{\varepsilon \cdot l}$$

The calculated concentrations are presented in Table 5.

As shown, the concentration of triiodide ions increased proportionally with increasing concentration of the D/PVA/I complex in the formulations. This observation confirms the concentration-dependent spectral response consistent with the Beer–Lambert relationship [40,42].

The obtained results indicate preservation of triiodide ions following incorporation of the D/PVA/I complex into the PEG matrix. Moreover, the proportional increase in absorbance and calculated triiodide ion concentration suggests the absence of significant degradation or transformation of iodine-containing species during formulation preparation.

Overall, the spectrophotometric analysis confirmed stability of the iodine–iodide system within the developed semisolid formulations and demonstrated reproducible incorporation of the active iodine-containing complex into the PEG-based matrix.

### 2.6. Calibration Curve of Triiodide Ion

To improve the accuracy of quantitative determination, a calibration relationship between absorbance and triiodide ion (I_3^−^_) concentration was established.

Standard solutions of triiodide ions were prepared by serial dilution of a stock iodine solution in the presence of excess potassium iodide (KI), which ensures the formation of a stable I_3^−^_ complex. The concentrations of the standard solutions ranged from (1–10) × 10^−6^ mol/L.

Absorbance was measured at approximately 350 nm using a quartz cuvette with an optical path length of 1 cm. A KI-containing solution was used as a blank.

Based on the obtained data, a calibration curve describing the dependence of absorbance on triiodide ion concentration was constructed. The relationship was found to be linear and is described by the following equation:

(3) A=k⋅c+b

where *k* is the slope of the calibration curve (method sensitivity) and *b* is the intercept (representing the absorbance of the blank or systematic deviation).

**Figure 5:**
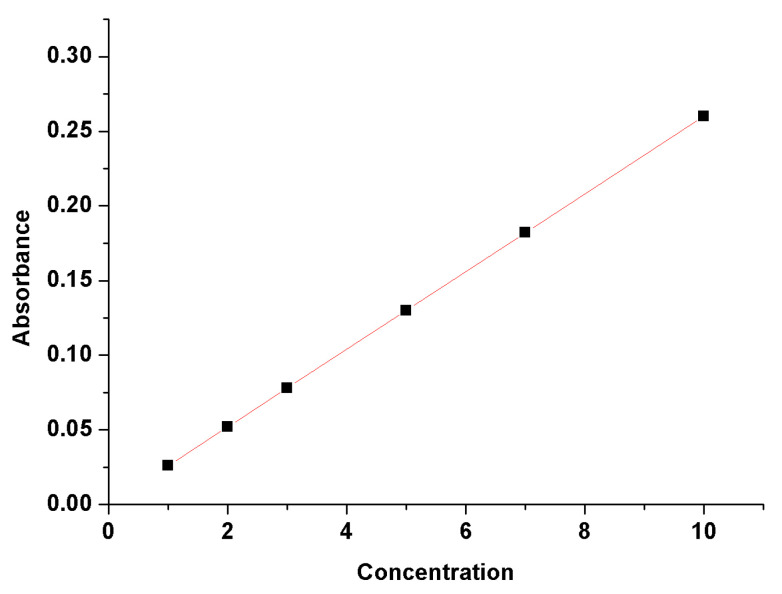
Calibration curve showing the dependence of absorbance on triiodide ion concentration.

The coefficient of determination (R^2^) was ≥0.99, confirming the applicability of the Beer–Lambert law within the studied concentration range (Table 6 and Figure 5).

The limit of detection (LOD) and limit of quantification (LOQ) for the spectrophotometric determination of triiodide ions were estimated according to ICH Q2(R1) guidelines. Based on the signal-to-noise approach using placebo baseline variability (σ ≈ 0.003 AU), the LOD (3.3σ/S) and LOQ (10σ/S) were calculated as 0.38 × 10^−6^ mol/L and 1.15 × 10^−6^ mol/L, respectively. The practical LOQ, defined as the lowest point of the linear calibration range, was 1.0 × 10^−6^ mol/L.

The obtained calibration relationship was subsequently used for determination of triiodide ion concentrations in the developed semisolid formulations. The calculated values demonstrated proportional increases in absorbance with increasing D/PVA/I content, confirming suitability of the spectrophotometric method for quantitative analysis of iodine-containing species.

### 2.7. Antimicrobial Activity

The antimicrobial activity of PEG-based hydrophilic ointment (semisolid) compositions containing the D/PVA/I complex was evaluated using the minimum bactericidal concentration (MBC) method and the agar diffusion assay against *Staphylococcus aureus* ATCC 6538P, *Escherichia coli* ATCC 8739, *Enterococcus hirae* ATCC 10541, and *Pseudomonas aeruginosa* ATCC 9027.

#### 2.7.1. Minimum Bactericidal Concentration (MBC)

The MBC values for the tested formulations are presented in Table 7.

At concentrations of 2.5% and 5.0%, the MBC values were 0.02 µg/mL for all tested strains. An increase in the D/PVA/I concentration to 10.0% resulted in a decrease in MBC values to 0.01 µg/mL, indicating enhanced antimicrobial activity.

These results demonstrate that the developed formulations exhibit strong antimicrobial activity against both Gram-positive (*S. aureus*, *E. hirae*) and Gram-negative (*E. coli*, *P. aeruginosa*) bacteria. No pronounced differences in susceptibility among the investigated strains were observed, suggesting broad-spectrum antimicrobial effects.

The absence of significant differences between the 2.5% and 5.0% formulations may indicate attainment of an effective antimicrobial concentration, whereas the lower MBC values observed for the 10.0% formulation may be associated with increased availability of active iodine-containing species.

#### 2.7.2. Agar Diffusion Assay

The results of the agar diffusion assay are presented in Table 8.

All investigated formulations demonstrated antimicrobial activity within the concentration range of 2.5–10.0%, as confirmed by inhibition zone diameters exceeding the well diameter (6 mm) for all tested strains.

Analysis of the concentration–effect relationship revealed partially non-linear behavior for several microorganisms. For *Staphylococcus aureus* and *Escherichia coli*, an increase in concentration to 5.0% resulted in enhanced antimicrobial activity, whereas a further increase to 10.0% led to a slight reduction in inhibition zone diameters.

For *Enterococcus hirae*, a significant increase in antimicrobial activity was observed when the concentration increased from 2.5% to 5.0%, followed by a plateau, suggesting the attainment of an effective concentration. In contrast, *Pseudomonas aeruginosa* exhibited relatively constant inhibition zone diameters across the tested concentration range, indicating the absence of pronounced dose dependence.

The absence of strict dose dependence in the agar diffusion assay may be associated with diffusion limitations of more concentrated semisolid systems within the agar medium.

#### 2.7.3. Comparative Analysis of Methods

The MBC data complemented the agar diffusion results and confirmed the pronounced intrinsic antimicrobial activity of the D/PVA/I complex. Whereas the agar diffusion assay reflects diffusion capacity of the formulation within a solid medium, the MBC method provides direct evaluation of antimicrobial activity in a liquid system.

Overall, the developed PEG-based hydrophilic semisolid formulations containing the D/PVA/I complex demonstrated pronounced antimicrobial activity against both Gram-positive and Gram-negative microorganisms. Under diffusion-controlled conditions, the 5.0% formulation demonstrated favorable antimicrobial performance, whereas the 10.0% formulation exhibited the lowest MBC values.

### 2.8. Cytotoxicity Evaluation

**Figure 6:**
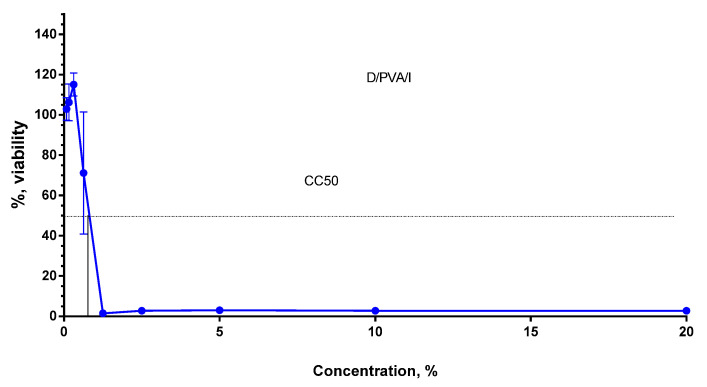
Cytotoxic effect of the D/PVA/I complex on MDCK cells.

**Figure 7:**
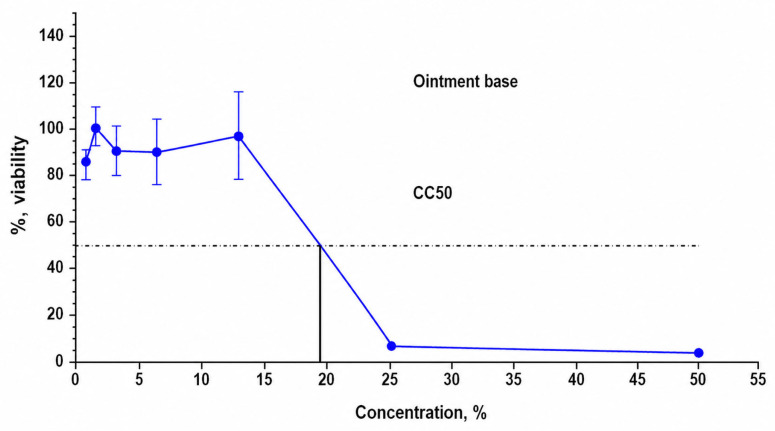
Cytotoxic effect of PEG-based hydrophilic ointment compositions on MDCK cells.

The cytotoxicity of the investigated samples was evaluated using the MTT assay in MDCK cell culture. Cell viability was assessed after 72 h of exposure to the tested samples, and *CC*_50_ values (the concentration reducing cell viability by 50%) were calculated based on the obtained data (Table 9; Figure 6 and Figure 7).

The results demonstrated a concentration-dependent decrease in MDCK cell viability for both the hydrophilic PEG-based ointment compositions containing the D/PVA/I complex and the ointment base.

The calculated *CC*_50_ values indicated that the D/PVA/I complex exhibits moderate cytotoxicity (*CC*_50_ = 0.82%), whereas the PEG-based ointment base (PEG 4000, PEG 400, and glycerin) showed a significantly higher safety threshold (*CC*_50_ = 18.38%), despite exhibiting mild cytotoxic effects at higher concentrations.

These findings suggest that the observed cytotoxicity is primarily associated with the active component (D/PVA/I), while the PEG-based matrix can be considered a relatively safe carrier system.

## 3. Discussion

The present study demonstrated that incorporation of the D/PVA/I complex into a PEG-based hydrophilic semisolid matrix resulted in formation of physically stable formulations with favorable physicochemical and biological characteristics. The obtained results highlight the important role of polymer–matrix interactions in modulation of iodine stability, rheological behavior, and antimicrobial activity [9,15,16].

The pH values of the developed formulations (4.94–5.45) remained within the physiologically acceptable range for topical semisolid systems, consistent with previous reports on PEG-based formulations [20,21,27]. The gradual decrease in pH with increasing D/PVA/I concentration may be associated with the presence of iodine-containing species and equilibrium processes involving iodide and triiodide ions within the polymeric matrix [9,39]. Importantly, the obtained pH values are considered compatible with skin application [34].

Rheological analysis demonstrated non-Newtonian pseudoplastic (shear-thinning) behavior for all investigated formulations. Such flow behavior is characteristic of hydrophilic semisolid systems and is advantageous for topical application because it facilitates spreading under mechanical stress while maintaining adequate consistency at rest [35,36,37]. Increased viscosity observed at higher D/PVA/I concentrations may be associated with enhanced intermolecular interactions and formation of a more structured internal polymeric network. Similar rheological behavior has been previously reported for polymer-based iodine-containing systems [9,16].

UV–visible spectroscopic analysis confirmed preservation of triiodide ions (I_3^−^_) within the PEG-based matrix, as evidenced by characteristic absorption maxima near 287 and 350 nm. Preservation of these absorption bands across all investigated formulations indicates that incorporation into the PEG matrix does not significantly alter the electronic structure of iodine-containing species. This observation is important because antimicrobial activity of iodine systems is closely related to availability of active iodine species in equilibrium with polyiodide forms [9,39]. Thus, the PEG-based matrix appears to provide a stabilizing environment for iodine-containing species while preserving their spectroscopic and biological activity [15].

Additional placebo control analysis further confirmed that the characteristic absorption bands observed in the active formulations were predominantly associated with triiodide ions rather than with the PEG ointment matrix. The placebo formulation demonstrated only weak nonspecific background absorption without distinct maxima near 287 and 350 nm. Minor spectral fluctuations observed in the 327–384 nm region in both placebo and iodine-containing formulations are likely related to nonspecific background absorption and light-scattering effects of the PEG-based matrix and auxiliary excipients. Importantly, these minor irregularities did not affect the clear detection of the characteristic triiodide absorption peaks. The obtained results therefore demonstrate that the developed PEG-based semisolid system maintains the structural stability and spectroscopic properties of iodine-containing species after incorporation into the ointment matrix.

Antimicrobial studies demonstrated activity of the developed formulations against both Gram-positive and Gram-negative microorganisms, including *Staphylococcus aureus*, *Escherichia coli*, *Enterococcus hirae*, and *Pseudomonas aeruginosa*. The obtained MBC values (0.01–0.02 µg/mL of active iodine) indicate pronounced intrinsic antimicrobial activity of the D/PVA/I system [4,5,15]. It should be noted that the agar diffusion assay did not reveal a strict linear relationship between the D/PVA/I concentration in the ointment and the diameter of the microbial growth inhibition zone. For *Enterococcus hirae*, increasing the ointment concentration from 2.5% to 5.0% was accompanied by a marked increase in the inhibition zone (from 10.33 to 17.33 mm), whereas further elevation to 10.0% did not result in additional enhancement of the effect. For *Escherichia coli*, the maximum inhibition zone diameter was observed at 5.0% (18.33 mm), with a slight reduction to 16.33 mm at 10.0%. For *Pseudomonas aeruginosa*, the inhibition zone diameters remained unchanged across the entire concentration range tested.

The absence of a linear concentration–response relationship can be attributed to methodological characteristics inherent to the agar diffusion assay [36,37]. The diameter of the inhibition zone is determined not only by the intrinsic antimicrobial activity of the substance but also by its ability to be released from the ointment base and diffuse through the agar medium. At higher concentrations of the active complex, the diffusion rate may be limited by the physicochemical properties of the semisolid composition—including increased viscosity and enhanced polymeric network density—leading to a plateau in the antimicrobial response. Furthermore, once the concentration exceeds the minimum inhibitory concentration (MIC) for a given microorganism, further increases in the active component content are not necessarily accompanied by a proportional expansion of the growth inhibition zone. These findings suggest that, for several tested strains, the maximal antimicrobial effect was already achieved at the 5.0% ointment concentration, and additional increases in D/PVA/I content did not provide a substantial improvement in activity.

Cytotoxicity studies were performed using the Madin–Darby Canine Kidney (MDCK, NBL-2, ATCC CCL-34) epithelial cell line, derived from canine kidney (Canis familiaris). The MDCK model was selected for this preliminary safety screening in accordance with ISO 10993-5 and OECD guidelines for in vitro cytotoxicity testing, offering high reproducibility and robust adherence properties for initial ranking of cytotoxic potential [28,29]. The D/PVA/I complex demonstrated moderate cytotoxicity (*CC*_50_ = 0.82%), whereas the PEG-based semisolid base exhibited substantially lower toxicity (*CC*_50_ = 18.38%), indicating that the observed biological effects are primarily associated with iodine-containing active species rather than the carrier matrix itself. At the same time, the relatively high *CC*_50_ value of the PEG-based matrix supports its suitability as a biocompatible carrier system [20,27]. Balancing antimicrobial efficacy and cytotoxicity represents a critical aspect in the development of topical iodine-containing formulations [5,6].

We acknowledge that MDCK cells, being of canine renal origin, are not fully representative of human skin architecture. For topical dermatological products, human keratinocyte cultures (e.g., HaCaT, NHEK) and reconstructed human epidermis (RHE) models provide superior translational relevance. Therefore, the present cytotoxicity data should be interpreted as a first-tier screening, and follow-up studies employing human skin-equivalent models are warranted to definitively establish the cutaneous safety profile of the developed formulations.

Overall, the obtained findings demonstrate that integration of physicochemical characterization, spectroscopic analysis, antimicrobial evaluation, and cytotoxicity assessment provides a comprehensive understanding of structure–property–activity relationships in PEG-based polymer–iodine semisolid systems. The developed formulations may therefore represent promising candidates for topical antimicrobial applications, particularly in the context of increasing antimicrobial resistance [1,2,4].

Future studies should focus on long-term stability evaluation, investigation of controlled release behavior under physiological conditions, and assessment of in vivo efficacy and safety. Additional optimization of formulation parameters may further improve the balance between antimicrobial activity and cytotoxicity, thereby enhancing therapeutic potential of the developed systems.

## 4. Materials and Methods

### 4.1. Materials

The dextrin/polyvinyl alcohol/iodine complex (D/PVA/I) was used as the active iodine-containing component. The complex was previously developed by the research group at JSC “Scientific Center for Anti-Infectious Drugs” (Almaty, Kazakhstan).

The following reagents and materials were used for preparation of the D/PVA/I complex: potato starch (Rogoznitsky Starch Plant, Lyada, Belarus, 98%), polyvinyl alcohol (molecular weight 31,000; Sigma-Aldrich, Darmstadt, Germany, 99.8%), hydrochloric acid, sodium hydroxide (JSC “Base No. 1 Chemreaktiv”, Staraya Kupavna, Russia, ≥99%), crystalline iodine, potassium iodide (G. Amphray Laboratories, Mumbai, India), and purified water.

Polyethylene glycol 400 (PEG 400), polyethylene glycol 4000 (PEG 4000), and glycerin were used as components of the hydrophilic semisolid base. PEG 400 and PEG 4000 were obtained from Sisco Research Laboratories Pvt. Ltd. (Mumbai, India) (PEG 400, lot No. 8378756; PEG 4000, lot No. 4584841). Glycerin was used as a humectant and plasticizing agent.

The combination of PEG 400, PEG 4000, and glycerin enabled formation of homogeneous hydrophilic semisolid formulations with suitable rheological properties.

All reagents used for microbiological and cell culture studies were of analytical grade and were used without additional purification.

### 4.2. Preparation of Hydrophilic Ointment Compositions

Hydrophilic semisolid formulations containing 2.5%, 5.0%, and 10.0% (*w*/*w*) of the D/PVA/I complex were prepared using a polyethylene glycol (PEG)-based matrix composed of PEG 4000, PEG 400, and glycerin.

PEG 4000 was melted at 60–65 °C, followed by addition of PEG 400 and glycerin under continuous stirring until formation of a homogeneous base. After cooling to approximately 40 °C, the required amount of the D/PVA/I complex was gradually incorporated under continuous mixing to ensure uniform distribution of the active component.

The formulations were stirred until homogeneous semisolid systems were obtained and subsequently equilibrated at room temperature prior to further analysis.

**Figure 8:**
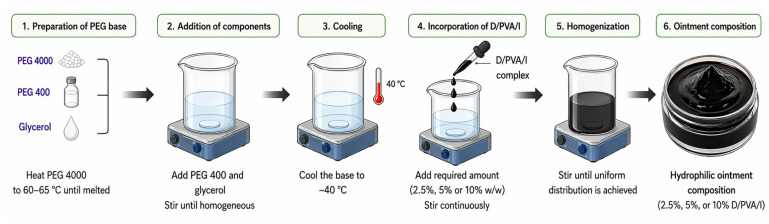
The process of obtaining hydrophilic ointment compositions based on PEG.

The preparation process of the PEG-based hydrophilic semisolid formulations containing the D/PVA/I complex is illustrated in Figure 8.

### 4.3. Physicochemical Characterization

#### 4.3.1. Organoleptic Properties and pH

Organoleptic properties of the developed formulations, including color, homogeneity, and consistency, were evaluated visually. The pH values were determined at room temperature using a calibrated pH meter with standard buffer solutions (Reagecon Diagnostics Ltd., Shannon, Ireland). All measurements were performed in triplicate.

#### 4.3.2. Rheological Analysis

Rheological properties were evaluated using a rotational digital viscometer (Brookfield-type viscometer, model HBDV-2, AMETEK Brookfield, Middleboro, MA, USA). Measurements were performed at rotational speeds ranging from 30 to 100 rpm under controlled temperature conditions.

Viscosity values were recorded, and flow behavior of the formulations was analyzed to determine rheological characteristics of the developed semisolid systems. Additionally, shear stress and shear rate data obtained from the viscometer were fitted to the Ostwald–de Waele power-law model (τ = Kγ^n^) to determine the flow behavior index (n) and consistency index (K). All measurements were performed in triplicate, and the results are presented as mean ± standard deviation (SD).

#### 4.3.3. UV–Visible Spectroscopy

UV–visible spectral analysis was performed in the wavelength range of 250–400 nm using a UV–visible spectrophotometer. Prior to analysis, samples were appropriately diluted. In addition to formulations containing the D/PVA/I complex, a placebo PEG-based hydrophilic ointment formulation without the iodine-containing active substance was also analyzed as a control to evaluate the possible contribution of the ointment matrix to the observed absorption profile. The presence of triiodide ions (I_3^−^_) was confirmed by characteristic absorption maxima near 287 nm and 350 nm.

### 4.4. Antimicrobial Activity

Hydrophilic ointment compositions containing the active ingredient (D/PVA/I) were evaluated at concentrations of 2.5%, 5.0%, and 10.0%.

Antimicrobial activity was assessed using two complementary methods: the serial dilution method to determine the minimum bactericidal concentration (MBC) and the agar well diffusion method.

#### 4.4.1. Determination of the Minimum Bactericidal Concentration (MBC)

The MBC was determined using a twofold serial dilution method in liquid medium. The samples were dissolved in sterile physiological saline (0.9% NaCl, pH 7.2) and mixed using a thermoshaker for 30 min at 750 rpm and 25 °C to ensure uniform dispersion.

Antimicrobial activity was evaluated against four standard strains: *Staphylococcus aureus* ATCC 6538P, *Escherichia coli* ATCC 8739, *Enterococcus hirae* ATCC 10541, and *Pseudomonas aeruginosa* ATCC 9027.

A series of twofold serial dilutions of the test samples was prepared. After incubation, aliquots from each dilution were plated onto agar media and incubated at 37 °C for 24 h. The MBC was defined as the lowest concentration at which no visible bacterial growth was observed.

Control samples consisted of culture media without the test formulation, inoculated with the same bacterial suspensions.

#### 4.4.2. Agar Well Diffusion Method

Antibacterial activity was also evaluated using the agar well diffusion method against the same test strains.

Bacterial suspensions at a concentration of 1.5 × 10^6^ CFU/mL were uniformly spread onto Mueller–Hinton agar (MHA) plates. Wells with a diameter of 6 mm were created using a sterile cylinder, and the test ointment formulations were carefully introduced into the wells.

The plates were incubated at 37 °C for 24 h, after which the diameters of the inhibition zones were measured.

### 4.5. Cell Culture and Cytotoxicity Analysis

#### 4.5.1. Cell Culture

The Madin–Darby canine kidney MDCK (epithelial cell line derived from canine kidney (*Canis familiaris*)) was used for cytotoxicity studies. Cells were obtained from RSE “Scientific Research Institute for Biological Safety Problems” (MDCK (NBL-2), ATCC. CCL-34). Cells were cultured in DMEM supplemented with 10% FBS and an antibiotic–antimycotic solution (100 U/mL penicillin, 0.1 mg/mL streptomycin, and 0.25 µg/mL amphotericin B) at 37 °C in a humidified atmosphere containing 5% CO_2_ [44,45].

#### 4.5.2. Determination of Cytotoxicity In Vitro

The in vitro cytotoxicity of the test samples was evaluated using the MTT assay [45]. Cells were seeded into 96-well plates at a density of 2.5 × 10^5^ cells/mL and incubated at 37 °C in a 5% CO_2_ atmosphere.

After 24 h of incubation, the growth medium was removed, and 200 µL of DMEM containing the test samples at the specified concentrations was added to each well. Negative control wells received 200 µL of incomplete DMEM medium.

Following 72 h of incubation, the medium was removed, and 200 µL of fresh growth medium along with 50 µL of MTT working solution was added to each well. The plates were incubated for an additional 4 h at 37 °C.

After incubation, the supernatant was carefully removed, and 100 µL of dimethyl sulfoxide (DMSO) was added to each well to dissolve the formazan crystals. Optical density was measured using a Tecan Sunrise RC.4 microplate reader (Tecan austria GmbH, Grödig, Austria) at a primary wavelength of 540 nm and a reference wavelength of 620 nm.

The results were calculated using Formulas (4)–(6):

The mean optical density (OD) of the negative control was calculated using Formula (4):

(4) $$\bar{Y}=\frac{y_{1}+\ldots +y_{n}}{n}=\frac{1}{n}\sum_{i=1}^{n}y_{i}$$

where Σ denotes the summation sign;

*y_i_* is the optical density (OD) value measured for each sample in the group;

*n* is the number of samples in the group.

The percentage of viable cells was calculated for each replicate at each concentration of the test sample using Formula (5):

(5) $$\%Viability=\frac{Y_{i}}{{\bar{Y}}_{NC}}\times 100\%$$

where *Y_i_* is a result of OD measuring for every object in the group;

${\bar{Y}}_{NC}$ is arithmetic mean value of OD for *NC*.

The *CC*_50_ (the concentration of test item at which 50% of cells die) for each test item was calculated using Formula (6):

(6) $$CC_{50}=\left[\frac{X1-50}{X1-X2}\times \left(Mx2-Mx1\right)\right]+Mx1$$

where *X*1 denotes more than 50% cell viability; *X*2 denotes less than 50% cell viability;

*Mx*1 is the concentration of the test item at which more than 50% of cells remain viable; and

*Mx*2 is the concentration of the test item at which less than 50% of cells remain viable.

#### 4.5.3. Statistical Analysis

Quantitative data were analyzed using one-way analysis of variance (one-way ANOVA). Statistical processing and graphical visualization were performed using GraphPad Prism 6 software.

## 5. Conclusions

PEG-based hydrophilic semisolid formulations containing the dextrin/polyvinyl alcohol/iodine complex (D/PVA/I) were successfully developed and comprehensively characterized. All formulations exhibited homogeneous semisolid structure, physiologically acceptable pH values, and pseudoplastic rheological behavior favorable for topical application.

UV–visible spectroscopic analysis confirmed preservation and stability of triiodide ions (I_3^−^_) following incorporation of the D/PVA/I complex into the PEG-based matrix. Antimicrobial evaluation demonstrated broad-spectrum activity against both Gram-positive and Gram-negative microorganisms, with low minimum bactericidal concentration values of 0.01–0.02 µg/mL (expressed as active iodine concentration).

Based on a comprehensive assessment of antimicrobial efficacy, rheological properties, and preliminary cytotoxicity data, the 5.0% D/PVA/I formulation is identified as the most balanced candidate. This concentration exhibited optimal antimicrobial performance in both liquid (MBC) and solid-phase (agar diffusion) assays, combined with favorable viscosity and spreadability characteristics. The 2.5% formulation, while displaying adequate antimicrobial activity, showed lower viscosity that may compromise retention at the application site, whereas the 10.0% formulation, despite enhanced intrinsic antimicrobial potency, was associated with increased matrix rigidity that may limit diffusion and ease of application. Cytotoxicity studies revealed moderate toxicity of the D/PVA/I complex (*CC*_50_ = 0.82%) and substantially lower toxicity of the PEG-based carrier matrix (*CC*_50_ = 18.38%), supporting acceptable preliminary biocompatibility.

It should be emphasized, however, that the designation of the 5.0% formulation as optimal is preliminary and based on the comparative endpoints evaluated in this study. Definitive optimization requires additional investigation of long-term physicochemical stability, in vitro iodine release kinetics under physiological conditions, cutaneous biocompatibility using human keratinocyte or reconstructed epidermis models, and comparative antimicrobial benchmarking against established iodine formulations (e.g., povidone–iodine). These directions have been prioritized for follow-up studies.

## Figures and Tables

**Table 1:** Organoleptic and physical characteristics of PEG-based hydrophilic ointment compositions.

D/PVA/I Concentration (%)	Appearance	Color	Odor	Homogeneity
2.5	Semisolid mass	Dark gray	Slight characteristic	Homogeneous
5.0	Semisolid mass	Black	Characteristic	Homogeneous
10.0	Semisolid mass	Black	Characteristic	Homogeneous

**Table 2:** pH values of PEG-based hydrophilic ointment compositions containing the D/PVA/I complex.

Concentration of D/PVA/I Complex (%)	pH
2.5	5.45
5.0	5.20
10.0	4.94

**Table 3:** Rheological parameters of hydrophilic ointments based on PEG according to the Ostwald–de Waele model.

Concentration of D/PVA/I Complex (%)	Flow Behavior Index (n)	Consistency Index (K, mPa·s^n^)	Flow Type
2.5	0.03	2.43 × 10^4^	Pseudoplastic
5.0	0.33	1.13 × 10^4^	Pseudoplastic
10.0	0.27	1.56 × 10^4^	Pseudoplastic

**Table 4:** UV–visible absorption maxima (λmax) and corresponding absorbance values of PEG-based hydrophilic ointment compositions containing different concentrations of the D/PVA/I complex.

Composition	Concentration of Complex D/PVA/I (%)	λmax_1_ (nm)	Optical Density	λmax_2_ (nm)	Optical Density
1	2.5	287.53	0.05	347.01	0.03
2	5.0	287.08	0.11	350.48	0.07
3	10.0	287.73	0.23	349.30	0.16

**Table 5:** Calculated triiodide ion concentrations in PEG-based hydrophilic ointment compositions.

Composition	Concentration of Complex D/PVA/I (%)	λmax_1_ (nm)	Optical Density	C (mol/L) ×10^−6^
1	2.5	287.53	0.05	1.92
2	5.0	287.08	0.11	4.23
3	10.0	287.73	0.23	8.85

**Table 6:** Data used for constructing the calibration curve of triiodide ion.

I_3^−^_ Concentration (×10^−6^ mol/L)	Optical Density (~350 nm)
1.0	0.026
2.0	0.052
3.0	0.078
5.0	0.130
7.0	0.182
10.0	0.260

**Table 7:** MBC values of PEG-based hydrophilic ointment compositions containing the D/PVA/I complex.

№	Names of Strains	D/PVA/I 2.5%	D/PVA/I 5%	D/PVA/I 10%
		Minimum Bactericidal Concentration, µg/mL		
1	*S. aureus* ATCC 6538P	0.02	0.02	0.01
2	*E. coli* ATCC 8739	0.02	0.02	0.01
3	*Ent. hirae* ATCC 10541	0.02	0.02	0.01
4	*P. aeruginosa* ATCC 9027	0.02	0.02	0.01

**Table 8:** Antimicrobial activity of PEG-based hydrophilic ointment compositions containing the D/PVA/I complex determined by the agar diffusion method.

Sample Name	Concentration, %	Growth Inhibition Zone, mm
		Average Value
*Staphylococcus aureus* АТСС 6538	2.5	16.33 ± 1.15
	5.0	17.0 ± 0.00
	10.0	18.0 ± 0.00
*Enterococcus hirae* АТСС 10541	2.5	10.33 ± 1.53
	5.0	17.33 ± 0.58
	10.0	17.33 ± 1.15
*Escherichia coli* АТСС 8739	2.5	18.0 ± 0.00
	5.0	18.33 ± 0.58
	10.0	16.33 ± 1.15
*Pseudomonas aeruginosa* АТСС 9027	2.5	17.0 ± 0.00
	5.0	17.0 ± 0.00
	10.0	17.0 ± 0.00

Note: The well diameter (Ø) was 6 mm.

**Table 9:** Cytotoxicity of the investigated samples in MDCK cell culture (*CC*_50_ values).

Test Item	*CC*_50_, [%], 72 h
D/PVA/I	0.82 ± 0.4
Ointment base	18.38 ± 1.3

## Data Availability

The original contributions presented in this study are included in the article. Further inquiries can be directed to the corresponding author.
